# A Deep-Sea Bacterium Is Capable of Degrading Polyurethane

**DOI:** 10.1128/spectrum.00073-23

**Published:** 2023-03-30

**Authors:** Zhi Gui, Guangchao Liu, Xin Liu, Ruining Cai, Rui Liu, Chaomin Sun

**Affiliations:** a Key Lab of Plant Biotechnology in Universities of Shandong Province, College of Life Science, Qingdao Agricultural University, Qingdao, China; b CAS and Shandong Province Key Laboratory of Experimental Marine Biology & Center of Deep Sea Research, Institute of Oceanology, Chinese Academy of Sciences, Qingdao, China; c Laoshan Laboratory, Qingdao, China; d Center of Ocean Mega-Science, Chinese Academy of Sciences, Qingdao, China; State Key Laboratory of Microbial Resources, Institute of Microbiology, Chinese Academy of Sciences

**Keywords:** polyurethane, degradation, deep sea, *Bacillus*, oxidoreductase

## Abstract

Plastic wastes have been recognized as the most common and durable marine contaminants, which are not only found in the shallow water, but also on the sea floor. However, whether deep-sea microorganisms have evolved the capability of degrading plastic remains elusive. In this study, a deep-sea bacterium Bacillus velezensis GUIA was found to be capable of degrading waterborne polyurethane. Transcriptomic analysis showed that the supplement of waterborne polyurethane upregulated the expression of many genes related to spore germination, indicating that the presence of plastic had effects on the growth of strain GUIA. In addition, the supplement of waterborne polyurethane also evidently upregulated the expressions of many genes encoding lipase, protease, and oxidoreductase. Liquid chromatography-mass spectrometry (LC-MS) results showed that potential enzymes responsible for plastic degradation in strain GUIA were identified as oxidoreductase, protease, and lipase, which was consistent with the transcriptomic analysis. In combination of *in vitro* expression and degradation assays as well as Fourier transform infrared (FTIR) analysis, we demonstrated that the oxidoreductase Oxr-1 of strain GUIA was the key degradation enzyme toward waterborne polyurethane. Moreover, the oxidoreductase Oxr-1 was also shown to degrade the biodegradable polybutylene adipate terephthalate (PBAT) film indicating its wide application potential.

**IMPORTANCE** The widespread and indiscriminate disposal of plastics inevitably leads to environmental pollution. The secondary pollution by current landfill and incineration methods causes serious damage to the atmosphere, land, and rivers. Therefore, microbial degradation is an ideal way to solve plastic pollution. Recently, the marine environment is becoming a hot spot to screen microorganisms possessing potential plastic degradation capabilities. In this study, a deep-sea *Bacillus* strain was shown to degrade both waterborne polyurethane and biodegradable PBAT film. The FAD-binding oxidoreductase Oxr-1 was demonstrated to be the key enzyme mediating plastic degradation. Our study not only provided a good candidate for developing bio-products toward plastic degradation but also paved a way to investigate the carbon cycle mediated by plastic degradation in deep-sea microorganisms.

## INTRODUCTION

It is estimated that about 1.15 to 2.41 million tonnes of plastic waste enter the oceans through rivers globally each year (1). Consequently, marine debris has become a worldwide problem that affects every region, continent, and coastal nation on Earth (2, 3). Most marine debris is not only found in the shallow water, but also on the sea floor (4). Plastic emissions from fishing and commercial shipping activities were the main source of deep-sea plastic pollution in the South China Sea (5). In addition to the oceans and coastal waters of the Pacific, Atlantic, and Indian Oceans, plastics and microplastics were now found in the South and Arctic and even in the Marianas Trench, the deepest part of the Earth’s surface (5–11). Plastic debris in the Mediterranean surface waters was dominated by millimeter-sized fragments (12). In deep waters, the abundance of microplastics varies between 2.06 and 13.51 pieces per liter, several times higher than that in open ocean groundwater. The abyssal zone may be one of the largest accumulations of microplastic debris on Earth (11). In the deep-sea environment, plastic pollution affected the growth, development, ability to avoid predators, and reproduction of marine organisms to varying degrees (5). Many studies reported that microplastics in water could be ingested by many marine organisms, such as zooplankton and fish (1). On the other hand, some marine microorganisms have evolved capabilities of degrading different plastics. For example, Takayoshi et al. isolated 13 different bacterial strains from the Kuril Islands and Japan trenches at depths of 5,000 to 7,000 m (the deeper ocean floor) as poly ε-caprolactone degrading bacteria. These strains have the potential to degrade aliphatic polyesters under deep-sea conditions, at low temperatures and high hydrostatic pressures (13).

Among various types of plastics, polyurethanes (PUR) are made from liquid isocyanates and liquid polyethers, and are widely used today. They are commercially available in a variety of forms, including flexible or rigid lightweight foams, ductile, rigid, and strong elastomers (14). They have been applied widely in the furniture, construction, footwear, and transport industries (15). Because most PURs are thermosetting polymers, they cannot be broken down easily under natural conditions and will remain in the environment for hundreds of years even longer (16). Moreover, its casual disposal inevitably pollutes the environment, and the landfill and incineration methods currently used cause serious damage to the atmosphere, land, and rivers (17, 18). To solve this problem, numerous PUR-degrading fungi have been isolated from a range of environments, and this has provided a large reservoir of microorganisms for the potential bioaugmentation of PUR waste (19–21). Moreover, several bacteria capable of degrading or transforming PUR have also been reported (22). These bacteria could generate transparent hydrolytic circles on the Impranil DLN containing plate (23), and could grow with Impranil DLN as the only carbon source (24), and almost completely degrade the added plastics when linear polyester PUR was used as the sole carbon source (25). However, until now, there is no commercial bio-product toward polyurethane degradation, which prompts researchers to find more powerful candidates for future application.

In this study, we used the waterborne polyurethane as the initial screening target and obtained a deep-sea bacterium Bacillus velezensis GUIA capable of degrading waterborne polyurethane. In combination with transcriptome and LC-MS analyses, we determined the FAD-binding oxidoreductase Oxr-1 as the key enzyme mediating plastic degradation. Oxr-1 was also able to degrade the PBAT film. Overall, our study provides not only a bacterial strain but also a promising enzyme candidate that could be used for development of biological products toward plastic degradation. Our study indicates that some deep-sea microbes might have evolved the ability to degrade plastics existing in the ocean, providing novel perspectives to study the carbon cycle mediated by plastic degradation in the deep biosphere.

## RESULTS AND DISCUSSION

### Isolation and identification of a deep-sea bacterium capable of degrading waterborne polyurethane.

**FIG 1:**
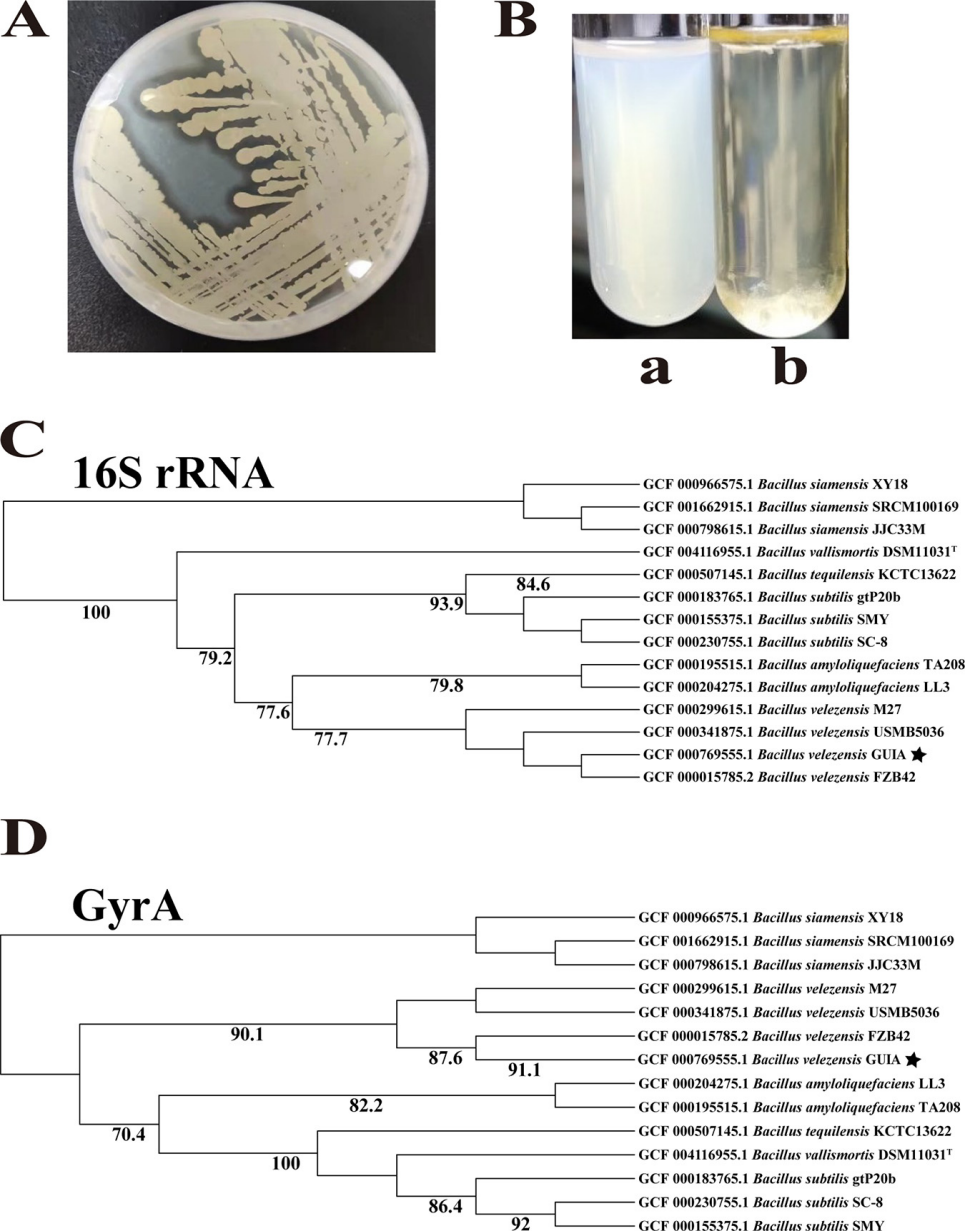
Identification of a deep-sea bacterium capable of degrading waterborne polyurethane. (A) Strain GUIA was capable of degrading waterborne polyurethane supplemented in the LB agar plate. 8 mL/L waterborne polyurethane was added in the medium. (B) Strain GUIA was capable of degrading waterborne polyurethane supplemented in the liquid medium. a: LB medium supplemented with 8 mL/L waterborne polyurethane; b: strain GUIA grown in LB medium supplemented with 8 mL/L waterborne polyurethane. (C) Phylogenetic tree based on 16S rRNA gene sequences of strain GUIA and related strains reconstructed by the Maximum likelihood method. Bootstrap values were based on 1,000 replicates (Bootstrap results are included and only those with bootstrap values >70% are shown). The black pentagram represents the evolutionary position of *B. velezensis* GUIA. (D) Phylogenetic tree based on partial GyrA protein sequences of strain GUIA and related strains reconstructed by the maximum likelihood method. Bootstrap values were based on 1,000 replicates (bootstrap results are included and only those with bootstrap values >70% are shown). The black pentagram represents the evolutionary position of *B. velezensis* GUIA.

Given that deep sea is a potential plastic-polluted environment in the present time, we were aiming to find out whether some microorganisms had evolved a capability of degrading plastic. With that, we initially chose waterborne polyurethane as the degradation target due to its some special characterizations, including high water solubility and easy degradation. Although detailed methods for detecting PUR degradation are inconsistent, waterborne polyurethane has always been the first choice to evaluate the degradation of polyurethane (26). To obtain microorganisms with the potential to degrade waterborne polyurethane, we spread the sedimental samples collected from the South China Sea on the 2216E agar plate supplemented with waterborne polyurethane (8 mL/L). Their abilities toward waterborne polyurethane degradation were evaluated by the appearance of clear zone around the colony (27). The clear zone method with agar plates is a widely used technique for screening plastic degrading bacteria and assessing the potential of different microorganisms for plastic degradation. It occurs when plastic degrading microorganisms secrete extracellular enzymes that diffuse through the agar and degrade the polymer into a water-soluble substance (27). After several rounds screening, we found a bacterial colony could produce an evident clear zone, indicating its potential of degrading waterborne polyurethane. This bacterium was then purified to a pure strain (named GUIA in this study) by routine microbiological methods, and its degradation capability toward waterborne polyurethane was verified in both agar plate and liquid medium supplemented with waterborne polyurethane. Indeed, strain GUIA could degrade the waterborne polyurethane and form clear zone around the bacterial colony (Fig. 1A). Moreover, strain GUIA could evidently degrade the waterborne polyurethane supplemented in the liquid medium, leading to a much clearer solution than that of control (Fig. 1B). Therefore, primary screening using waterborne polyurethane and secondary screening using other specific substrates are feasible in identifying PUR-degrading microorganisms (28).

In addition, 16S rRNA gene sequence (accession no. OP897227) and GyrA (gyrase) (29) protein sequence (accession no. NZ_CP009679.1) of strain GUIA showed high homology (higher than 99% identity) with those in the identified B. velezensis FZB42 strain (30), and clustered to the same branch in the 16S rRNA (Fig. 1C) and GyrA (Fig. 1D) phylogenetic trees. The above results strongly suggested that strain GUIA should be a member of B. velezensis. We thus designated this strain as B. velezensis GUIA in this study. *Bacillus* members are a kind of Gram-positive aerobic bacteria belonging to the family *Bacillariophyceae*. They are considered common members of the microbial community and occupy many environments on Earth. Indeed, a variety of *Bacillus* strains were reported to have degradation effect on polyurethane (31–33). B. velezensis GUIA, isolated from the deep-sea cold seep, was capable of degrading waterborne polyurethane. We therefore speculated that some deep-sea *Bacillus* strains have evolved abilities of plastic degradation and might be good candidates for developing bio-products toward plastic degradation.

To obtain the genetic basis of polyurethane degradation in *B. velezensis* GUIA, its whole genome was sequenced and analyzed. The basic genome information of strain GUIA is shown in Table S1 and Fig. S1. Only one chromosome was detected in the genome of strain GUIA. The full-length of the genome was 3,929,584 bp, GC content was 46.5%, and the predicted number of genes was 4,024. To further evaluate its plastic degradation capability, genes related to plastic biodegradation mentioned in previous studies (19, 34, 35) were conducted a detailed search within the genome of strain GUIA. As a result, a large number of genes encoding esterase, protease, and lipase were found. Previously, some researchers reported the degradation of polyurethane by esterase (36, 37); Howard et al. purified a 29 kDa protease from the strain Pseudomonas fluorescens that could cleave the urea bond of the amino-methyl unit in PUR (34); Howard et al. purified a 66 kDa lipase from strain Acinetobacter gerneri P7, which hydrolyzed the ester bond in polyester PUR (38); Russell et al. identified a 21 kDa serine hydrolase from the fungus *Pestalotiopsis microspora* E2712A that cut the urea bond of the amino-methyl unit in PUR (39). Therefore, combined with previous reports and the present experimental fact that the strain GUIA could degrade plastic, we speculated that these genes might be involved in its plastic degradation.

### Transcriptome analysis of *B. velezensis* GUIA incubated in the medium supplemented with waterborne polyurethane.

**FIG 2:**
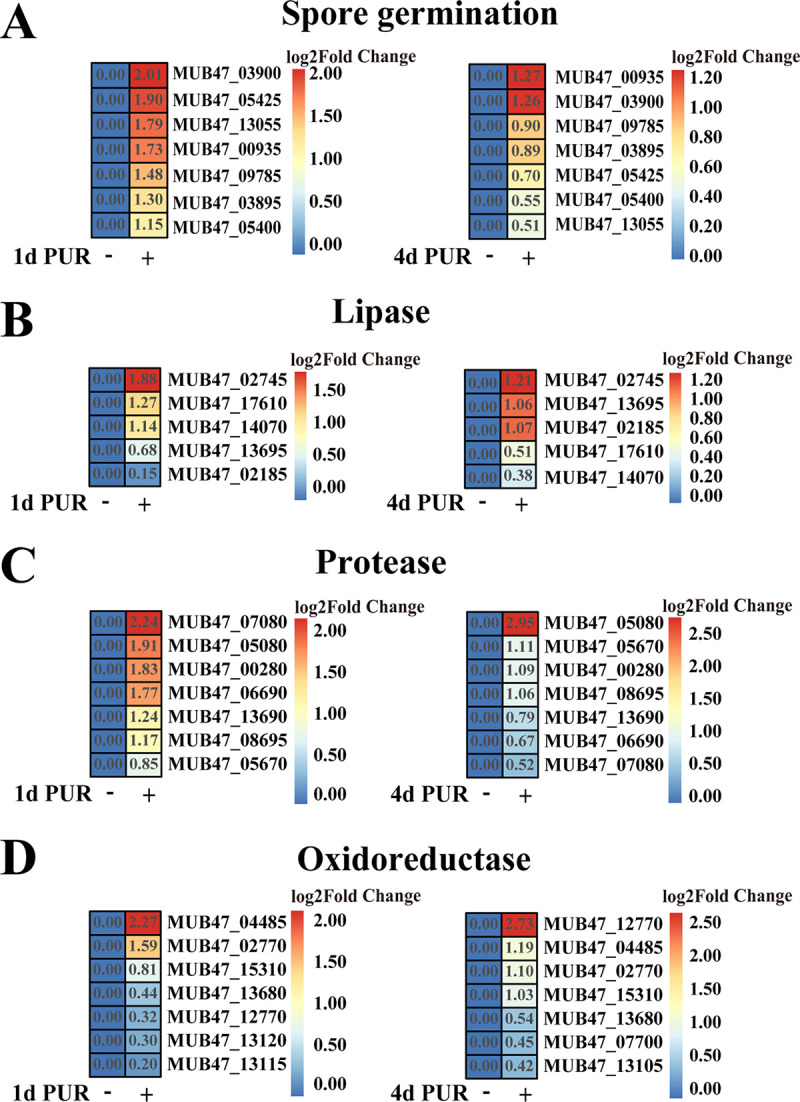
Transcriptomic analysis of *B. velezensis* GUIA cultured in the medium supplemented with waterborne polyurethane. (A) Transcriptomics based on heat map showing all upregulated genes associated with spore germination. (B) Transcriptomics based on heat map showing all upregulated genes encoding lipase. (C) Transcriptomics based on heat map showing all upregulated genes encoding protease. (D) Transcriptomics based on heat map showing all upregulated genes encoding FAD-binding oxidoreductase. In this figure, “+” indicates strain GUIA cultured in the LB medium added with 8 mL/L waterborne polyurethane; “-” indicates strain GUIA cultured in the LB medium only; “1d PUR” indicates the increasing times of expression of corresponding gene in the presence of 8 mL/L waterborne polyurethane after 1-day incubation. “4d PUR” indicates the increasing times of expression of corresponding gene in the presence of 8 mL/L waterborne polyurethane after 4-day incubation.

To explore the potential degradation process of waterborne polyurethane mediated by strain GUIA, we performed a transcriptome analysis of this strain cultured in the medium supplemented either with or without waterborne polyurethane for 1 and 4 days. The results showed that the presence of waterborne polyurethane had some effects on the growth of strain GUIA: on both 1 and 4 days incubation, the expression of many genes related to spore germination was upregulated (Fig. 2A). In addition, compared to absence of waterborne polyurethane, the expression of many genes encoding lipase (Fig. 2B) and protease (Fig. 2C) was evidently upregulated, which was consistent with previous reports that lipase and protease were potential enzymes responsible for polyurethane degradation (35, 38, 40). On the other hand, the expression of some genes encoding oxidoreductase was also upregulated (Fig. 2D). To our knowledge, oxidoreductase was not recognized as a common enzyme capable of degrading polyurethane, although this kind of enzyme had been proposed to possess degradation potentials toward other types of plastics (40, 41).

### Purification and identification of extracellular enzymes possessing potentials of degrading waterborne polyurethane.

**FIG 3:**
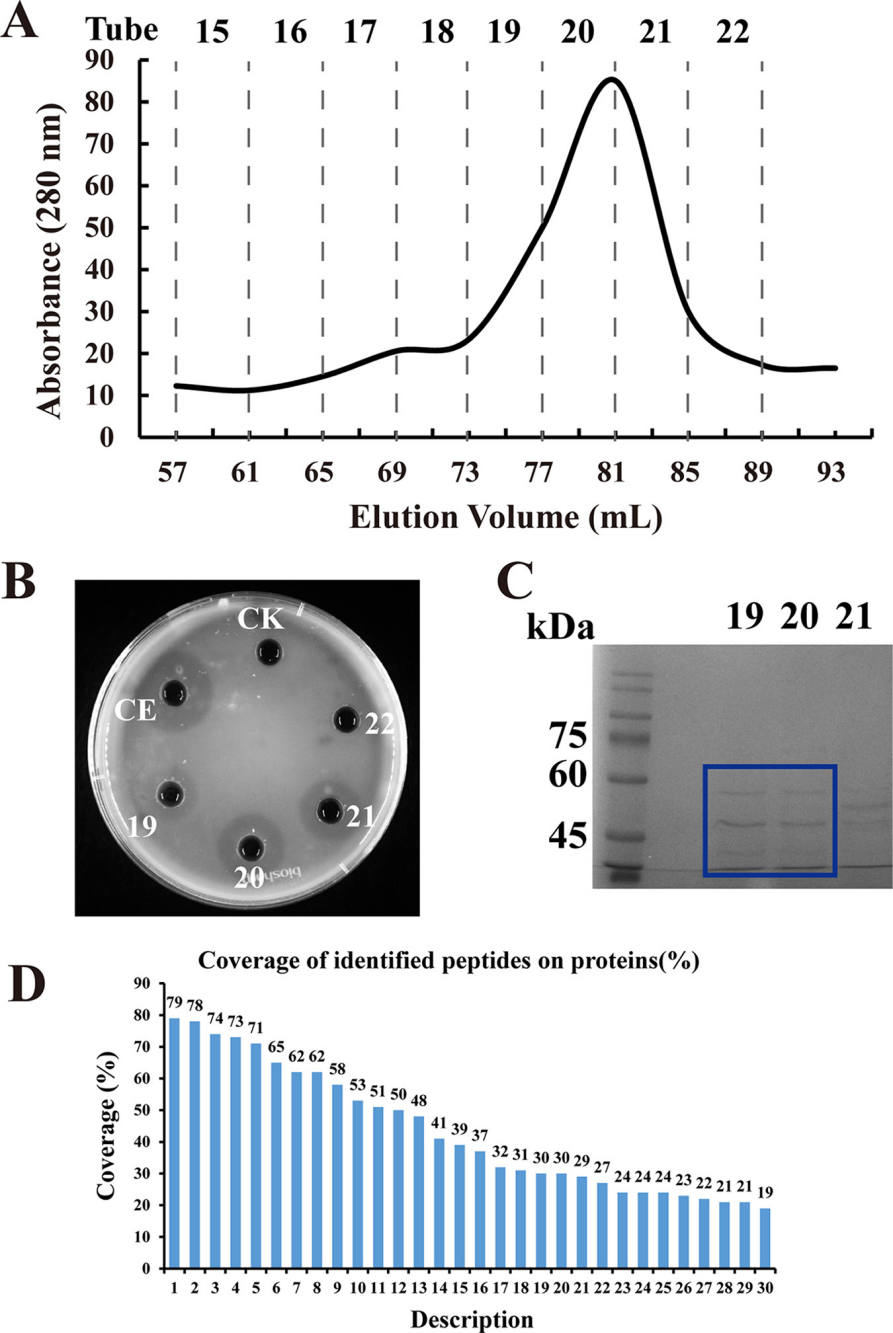
Purification and identification of proteins possessing potential degradation function toward waterborne polyurethane. (A) Purification chromatogram of extracellular proteins of strain GUIA that cultured in the LB medium supplemented with 8 mL/L waterborne polyurethane by the Superdex 200 column. (B) Functional analysis of purified extracellular proteins with degradation potentials toward waterborne polyurethane. CK, elution buffer; CE, extracellular crude enzymes extracted from strain GUIA; 19 to 21 represent fractions numbers based on the purification chromatogram in panel A. (C) SDS-PAGE analysis of extracellular proteins of strain GUIA with the degradation capability toward waterborne polyurethane. Numbers 19 to 21 represent fractions shown in panels A and B. The blue color rectangle indicates the area collected for liquid chromatography-mass spectrometry (LC-MS) analysis shown in panel D. (D) Identification of proteins with degradation potentials toward waterborne polyurethane through LC-MS. *y* axis represents the coverage of identified peptides on proteins (%). 1 to 30 in the *x* axis represent the protein names identified by LC-MS as following. 1, FAD-binding oxidoreductase; 2, A beta-lactamase; 3, glycoside hydrolase family 18 protein; 4, alanine racemase; 5, dihydrolipoyl dehydrogenase; 6, methionine ABC transporter substrate-binding lipoprotein MetQ; 7, right-handed parallel beta-helix repeat-containing protein; 8, glycerophosphodiester phosphodiesterase; 9, gamma-glutamyltransferase; 10, SGNH/GDSL hydrolase family protein; 11, outer spore coat protein CotE; 12, sporulation hydrolase CotR; 13, dihydrolipoyl dehydrogenase; 14, flagellar basal body rod protein FlgG; 15, heme-dependent peroxidase; 16, superoxide dismutase; 17, enoyl-ACP reductase FabI; 18, multifunctional 2'3′-cyclic-nucleotide 2′-phosphodiesterase/3′-nucleotidase/5′-nucleotidase; 19, arginase; 20, hypothetical protein; 21, 4-hydroxy-tetrahydrodipicolinate synthase; 22, SPFH domain-containing protein; 23, S8 family peptidase; 24, hydrolase; 25, RDD family protein; 26, 3-hydroxyacyl-ACP dehydratase FabZ; 27, purine-nucleoside phosphorylase; 28, carboxypeptidase M32; 29, SDR family oxidoreductase; 30, alanine racemase.

Next, we sought to find out which enzymes were indeed responsible for polyurethane degradation in strain GUIA. Logically, the functional degradation enzymes should be extracellular proteins. Therefore, we obtained the total extracellular proteins by ammonium sulfate precipitation and then separated them by molecular weight difference via a gel filtration column (Hiload 16/600 Superdex 200). Meanwhile, we checked the degradation activity of each fraction by observation of the clear zone shown in the agar plate supplemented with waterborne polyurethane. The functional assay results indicated that three fractions (tubes 19, 20, and 21; Fig. 3A) after gel filtration column showed obvious degradation activity (Fig. 3B). Based on the prediction of protein molecular weight by the calibration curve of gel filtration column (Fig. S2), we speculated that the molecular weight of the functional enzyme should be around 45 kDa. However, when the components of fractions 19 to 20 were checked through the SDS-PAGE gel (Fig. 3C), there were several bands around the molecular marker of 45 kDa, indicating that the content of each functional fraction was a mixture. To figure out the components of the fractions 19 and 20, we collected the bands around 45 kDa and performed the liquid chromatography-mass spectrometry (LC-MS) analysis. The LC-MS results showed that the major identified fragments matched well with proteins annotated as oxidoreductase, protease, lipase, and spore germination (Fig. 3D), which was consistent well with the transcriptomic analysis (Fig. 2). Together, we proposed that the real degradation enzyme in strain GUIA undoubtedly belongs to those types of enzymes shown in LC-MS and transcriptomic results.

### The oxidoreductase Oxr-1 of strain GUIA was demonstrated to be the key degradation enzyme toward waterborne polyurethane.

**FIG 4:**
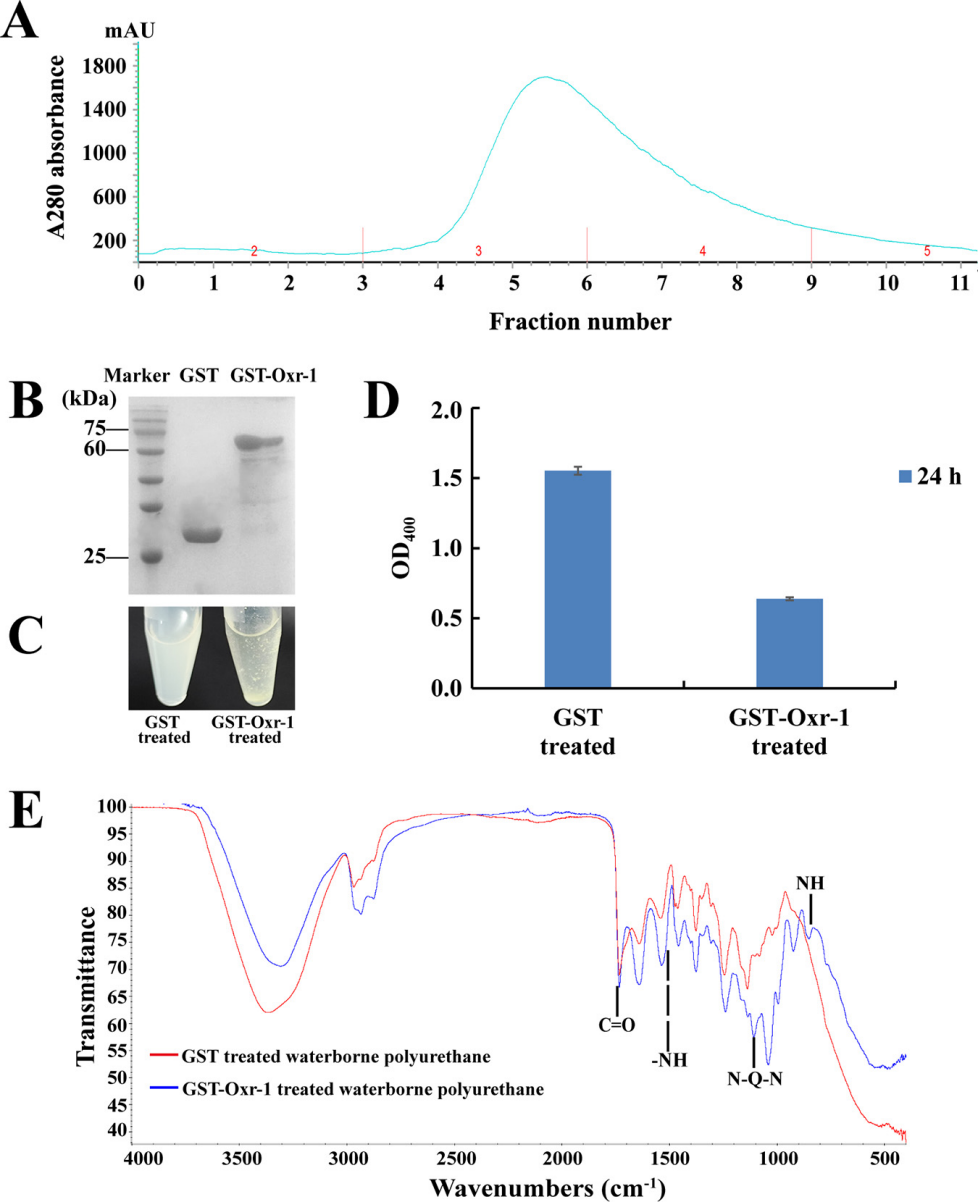
An FAD-binding oxidoreductase (Oxr-1) of strain GUIA was identified as the key enzyme responsible for degradation of waterborne polyurethane. (A) Purification chromatogram of GST fused Oxr-1 with the GST affinity column. (B) SDS-PAGE analysis of purified GST and GST-Oxr-1. The purified enzyme was electrophoresed on a 12% SDS-PAGE gel. (C) Degradation assay of GST-Oxr-1 toward waterborne polyurethane. GST was used as the control. (D) Quantitative assay of the degradation effect of GST-Oxr-1 and GST on waterborne polyurethane shown in panel C. Changes of OD_400_ of the waterborne polyurethane solution treated by GST and GST-Oxr-1 for 24 h were, respectively, measured. All data are represented by the mean ± standard deviation (SD). The final concentration of GST or GST-Oxr-1 used in each reaction was 1.25 mg/mL. (E) FTIR analysis of the degradation effect of GST-Oxr-1 on waterborne polyurethane. The peak of 1510 cm^−1^ was -NH, and 1124 cm^−1^ was identified as quinone. The structure of quinone was N-Q-N (Q was the characteristic vibration of quinone ring) in quinone ring.

Combined with previous (42) and present results, we first chose some proteases and lipases for *in vitro* overexpression and checked their degradation capabilities toward waterborne polyurethane. However, after much effort, we failed to find any degradation potential by using proteases and lipases indicated in both LC-MS and transcriptomic results. Therefore, we reanalyzed the results of LC-MS and transcriptome, the FAD-binding oxidoreductase (Oxr-1) (accession no. UOM43036.1) caught our attention. The expression of Oxr-1 was significantly upregulated in the presence of waterborne polyurethane (Fig. 2D), and it showed the highest coverage rate within all the identified proteins based on the LC-MS assay. We next sought to ask whether Oxr-1 is indeed the key degradation enzyme toward waterborne polyurethane for strain GUIA. With that, we overexpressed Oxr-1 in the Escherichia coli BL21(DE3) cell line (Fig. 4A), and it showed a high purity on the SDS-PAGE gel (Fig. 4B). As expected, Oxr-1 showed a good degradation effect on waterborne polyurethane (Fig. 4C), and it could decrease the amount of waterborne polyurethane to one third of that detected in the control after 24-h treatment (Fig. 4D).

To further understand the degradation process of polyurethane by Oxr-1, we checked the structure changes of waterborne polyurethane after treatment through Fourier transform infrared (FTIR). FTIR analysis showed that the functional groups between 1,732 cm^−1^ -851 cm^−1^ had significant changes. The peak of 1,510 cm^−1^ was indicated as -NH and 1,124 cm^−1^ was quinone, and the structure was proposed to be N-Q-N (Q was the characteristic vibration of quinone ring) in quinone ring (Fig. 4E) (43). There were several obvious changes in the FTIR chromatograph after Oxr-1’s treatment; however, we could not accurately infer the structure changes of waterborne polyurethane due to the its unknown structure information. Therefore, we concluded that Oxr-1 was one of the key enzymes responsible for degradation toward waterborne polyurethane in strain GUIA.

### The oxidoreductase Oxr-1 also degraded the biodegradable plastic.

**FIG 5:**
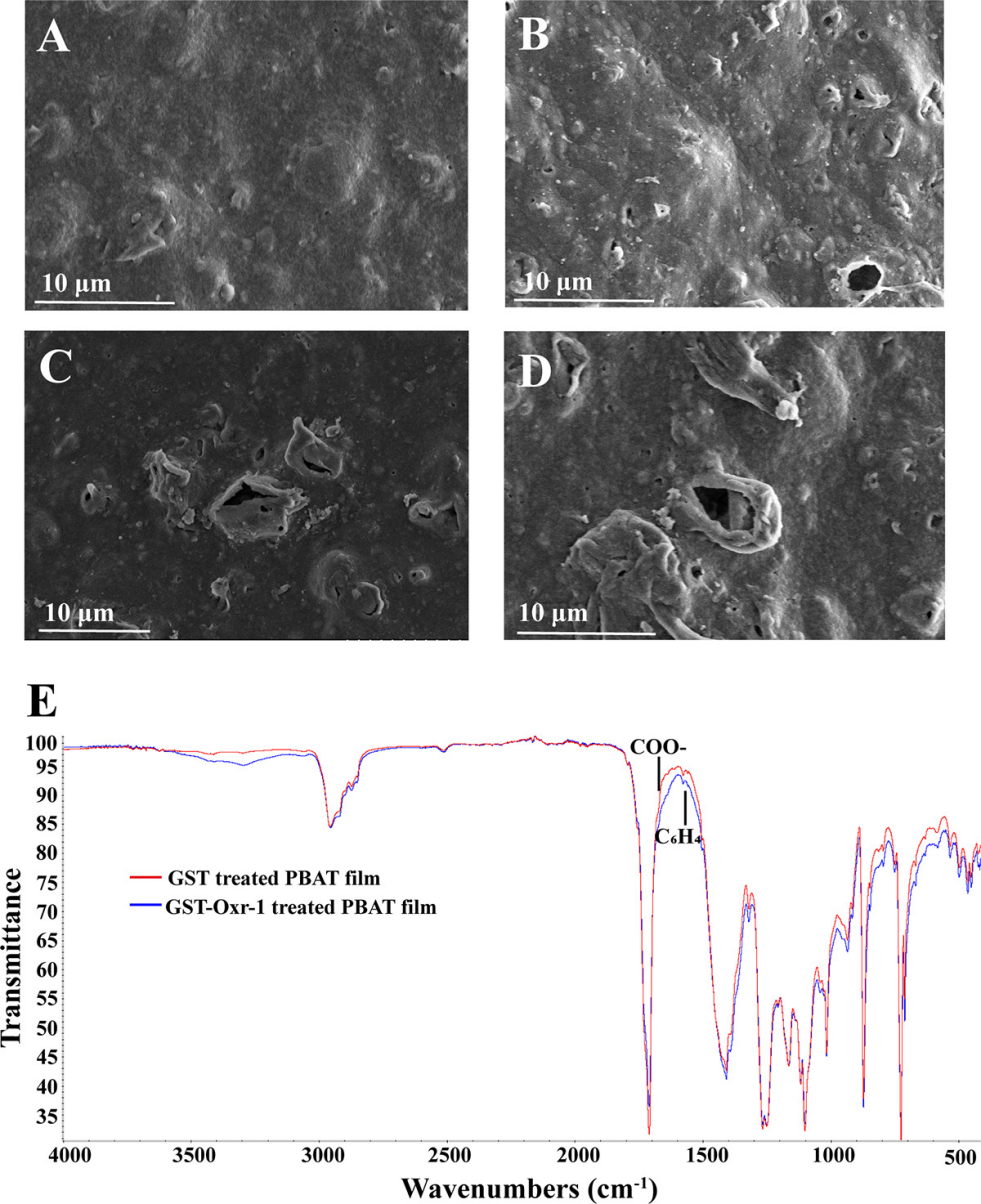
SEM and FTIR analyses of the degradation effect of Oxr-1 on the PBAT film. (A) SEM observation of the degradation effect of GST on the PBAT film for 24 h. (B to D) SEM observation of the degradation effect of the PBAT film treated by GST-Oxr-1 for 24 h. (E) FTIR analysis of the degradation effect of GST and GST-Oxr-1 on the PBAT film. The characteristic ester absorption peaks at 1,690 cm^−1^ indicated signs of degradation. In the FTIR spectrum of PBAT, the absorption band at 1,456 cm^−1^ is the characteristic group stretching of phenylene group, and it is an important sign of plastic degradation.

With the increase of plastic waste existing in the nature, more and more countries are choosing to use biodegradable plastic in routine life (44). However, the public should be clear that in many conditions, the biodegradable plastic must be recycled and fully degraded indoors. Otherwise, the biodegradable plastic will become another kind of plastic pollution (11). The biodegradable plastic mainly composed of PBAT is one of the most popular plastics used in the world (45). We next sought to check the degradation effect of Oxr-1 on the PBAT plastic to see whether it could be used for developing the pretreatment product toward PBAT degradation. To this end, we treated the PBAT film and then observed its morphology changes via scanning electron microscopy (SEM). The observation results showed that the treatment by Oxr-1 caused significant morphological changes in the film surface (Fig. 5B to D) compared with that of control (Fig. 5A). In particular, we observed numerous holes on the PBAT film surface and even in the deep inside of the film.

FTIR analysis was further carried out to explore the structure changes after 24-h treatment by Oxr-1. Changing patterns of absorption bands of treated PBAT film implied a change in their chemical structure (46) (Fig. 5E). For example, the characteristic ester absorption peaks at 1,690 cm^−1^ indicated signs of degradation; in the FTIR spectrum of PBAT, the absorption band at 1,456 cm^−1^ is the characteristic group stretching of phenylene group, and it is an important sign of plastic degradation (47).

Overall, we concluded that the deep-sea bacterium *B. velezensis* GUIA possessed a promising potential in degrading waterborne polyurethane. Different with previous reports, protease and lipase existing in this strain were not functional in degrading waterborne polyurethane. Unexpectedly, the oxidoreductase Oxr-1 identified in this strain was demonstrated to play a key role toward polyurethane degradation. Oxr-1 also degraded the biodegradable plastic to some extent. Our study not only provided a good candidate for developing bio-products toward plastic degradation but also paved a way to investigate the carbon cycle mediated by plastic degradation in deep-sea microorganisms. Future efforts will be required to explore the degradation mechanism of Oxr-1 and find out its effective mutant with higher degradation efficiency.

## MATERIALS AND METHODS

### Isolation of deep-sea microorganisms with potential degradation capability toward waterborne polyurethane.

Deep-sea sediments were collected from a typical cold seep (at a depth of 1,146 m) by *RV KEXUE* in July of 2018 as previously described (48). Deep-sea bacteria were isolated on the 2216E (5 g tryptone and 1 g yeast extract in 1 L filtered seawater and 15 g/L agar, pH adjusted to 7.4 to 7.6) agar plates at 28°C as described previously (49). To screen deep-sea bacteria possessing degradation potential toward polyurethane, bacterial cells were cultivated on the Luria-Bertani (LB) agar plate (10 g/L peptone, 5 g/L yeast extract, 10 g/L NaCl, and 15 g/L agar, pH adjusted to 7.0) supplemented with polyurethane emulsion (Shenzhen Yoshida Chemical Co., Ltd., No. F0402, 35% solids ± 5) (8 mL/L). The agar plates were incubated at 28°C for 1 to 2 days, and colonies with hydrolytic circles were selected for further study. Bacterial 16S rRNA gene was amplified by universal primers 27F (5′-AGAGTTTGATCCTGGCTCAG-3′) and 1492R (5′-TACGGCTACCTTGTTACGACTT-3′). Phylogenetic analysis of 16S rRNA sequences and GyrA protein sequences were performed by using the Basic Local Alignment Search Tool (BLAST) algorithm and MEGA software (50).

### Genome sequencing and analysis.

Genomic DNAs of strain GUIA were extracted from cells cultured for 24 h at 28°C in LB medium. The DNA library was prepared using the Ligation Sequencing Kit (SQK-LSK109), and genomic DNAs were sequenced by using a FLO-MIN106 vR9.4 flow-cell and MinKNOWN software v1.4.2 (Oxford Nanopore Technologies, United Kingdom). Whole-genome sequence determinations were carried out with the Oxford Nanopore MinION (Oxford, United Kingdom) and Illumina MiSeq sequencing platform (San Diego, USA). A hybrid approach was utilized for genome assembly using reads from both platforms. Base-calling was performed using Albacore software v2.1.10 (ONT). Nanopore reads were processed using protocols toolkit for quality control and downstream analysis (51). Filtered reads were assembled using Canu version 1.8 with the default parameters for Nanopore data (52). And then the genome was assembled into a single contig and was manually circularized by deleting an overlapping end.

### Transcriptome analysis.

Transcriptomic analysis was performed by Novogene (Tianjin, China). Strain GUIA cultured in LB medium supplemented with waterborne polyurethane (8 mL/L) for 1 and 4 days was used as the experimental group. At the same time, strain GUIA cultured in LB medium supplemented without waterborne polyurethane for 1 and 4 days was used as the control group. Afterwards, bacterial cultures were centrifuged at 8,000 *g* for 20 min at 4°C. Then, the cells were collected, washed three times with 10 mM sterile PBS buffer (137 mM NaCl, 2.7 mM KCl, 10 mM Na_2_HPO_4_, 1.8 mM KH_2_PO_4_, 1 L sterile water, pH 7.4), and used for transcriptome sequencing. Extraction of total RNAs from strain GUIA with TRIzol reagent (Invitrogen, USA) for transcriptome analysis and DNA contamination was removed using MEGA clear Kit (Life Technologies, USA), and RNA degradation and contamination were monitored on 1% agarose gel. The purity of RNA was examined using the NanoPhotometer spectrophotometer (Implen, USA). RNA concentration was measured using the Qubit RNA assay kit (Life Technologies, USA). RNA integrity was assessed using the Bioanalyzer 2100 System's RNA Nano 6000 detection kit (Agilent Technologies, USA). Sequencing libraries were generated using the NEBNext Ultra RNA Library Prep Kit for Illumina (NEB, USA) as recommended by the manufacturer, and indexing codes were added to assign sequences to each sample. The clustering of index coded samples was performed on the cBot cluster generator. After clustering generation, the preparation of the library was sequenced on the Illumina Hiseq platform and produced paired end-readings of 125 bp/150 bp. Raw data in fastq format (raw readings) was first processed by an internal perl script (53). The Reference Genome and Gene Model annotation files were downloaded directly from the Genome website. The reference genome was indexed using Hisat2 (v2.0.4), and the clean reading at the paired end was compared to the reference genome using Hisat2 (v2.0.4). HTSeq (v0.9.1) was used to calculate the readings mapped to each gene. The FPKM for each gene was then calculated based on the length of the gene and the reading mapped to that gene. Differential expression analysis was performed by gene ontology (GO) enrichment analysis using the DESeq R software package (v1.18.0) which was implemented by the GOseq R software package, where the gene length bias was corrected (54). KOBAS software examined the statistical enrichment of differentially expressed genes in the KEGG pathway (55). PPI analysis of differentially expressed genes was based on STRING database. The Cufflinks (v2.1.1) Reference Annotation Based Transcript (RABT) assembly method was used to construct and identify both known and novel transcripts from TopHat alignment results. Picard-tools (v1.96) and sam tools (v0.1.18) were used to sort, mark duplicated reads, and reorder the bam alignment results of each sample. GATK2 (v3.2) software was used to perform SNP calling.

### Purification of extracellular proteins with potential degradation capability toward waterborne polyurethane from strain GUIA.

Strain GUIA was inoculated in 1 L LB broth with the addition of 8 mL/L waterborne polyurethane and incubated at 28°C and 150 rpm for 4 days. After incubation, the extracellular proteins of strain GUIA were purified as described previously with minor modification (56). Cell-free supernatant was obtained after centrifugation at 10,000 *g* for 20 min, and then precipitated by 80% saturation with (NH_4_)_2_SO_4_ at 4°C overnight. The precipitate was collected by centrifugation and dissolved in 50 mM NaCl (20 mM Tris-HCl, pH 8.0) buffer and dialyzed against the same buffer overnight to obtain the crude extracellular proteins extract. This was then purified by a 5 mL HiTrapTM Q HP column (GE Healthcare, USA) with gradient elution buffer from 150 mM to 2 M NaCl in 20 mM Tris-HCl (pH 8.0) on an AKTA purifier system (Amersham Biosciences, USA). The degradation capability of each fraction was measured by the method described above to monitor the potential extracellular degradation proteins of strain GUIA. Then ultrafiltration (3 kDa MW interception membrane, Millipore) was used to concentrate the hydrolyzed active component, and subjected to gel filtration on a Superdex 200 column (GE Healthcare, USA) after pre-equilibrated by 150 mM NaCl with 20 mM Tris-HCl (pH 8.0). The purified active fractions were eluted with the same buffer at a flow rate of 1 mL/min, and the active fractions were used for further analysis. All purification processes were performed at 4°C. The protein content of each chromatographic fraction was determined by measuring the absorbance at 280 nm using an AKTA purifier system. The most active fraction was determined by sodium dodecyl sulfate-polyacrylamide gel electrophoresis (SDS-PAGE), which was performed on a 5% stacking and a 12% running gel according to the methods of Laemmli, then stained with Coomassie brilliant blue R250 (Rui Bo Xing Ke, China) (57).

### Liquid chromatography-mass spectrometry analysis.

To identify the components of above purified proteins targeting waterborne polyurethane degradation from strain GUIA, LC-MS analysis was performed. After centrifugation and drying, the enzyme-cleavage peptide samples were redissolved in Nano-LC mobile phase A (0.1% formic acid/water) and bottled for on-line LCMS analysis. The dissolved samples were placed on nanoViper C18 precolumn (3 μm, 100 A) at a volume of 2 μL, and then rinsed and desalinated at a volume of 20 μL. The liquid phase was Easy nLC 1200 nanoliter liquid phase system (Thermo Fisher Scientific, USA). The samples were desalted and retained on the precolumn and then separated by the analytical column. The analytical column was C18 reverse-phase column (Acclaim PepMap RSLC,75 μm × 25 cm C18-2 μm 100 A). The gradient of mobile phase B (80% acetonitrile, 0.1% formic acid) was increased from 5% to 38% within 30 min. Mass spectrometry was performed by Thermo Fisher Q Exactive system (Thermo Fisher Scientific, USA) combined with Nano Flex ion source (Thermo Fisher Scientific, USA). The spray voltage was 1.9 kV and the heating temperature of ion transmission tube was 275°C. The scanning mode of mass spectrometry was information dependent collection mode (DDA, data dependent analysis). The scanning resolution of primary mass spectrometry was 70,000, the scanning range is 350 to 2,000 *m/z*, and the maximum injection time was 100 ms. Under each DDA cycle, a maximum of 20 secondary spectra with charges from 2+ to 5+ were collected, and the maximum implantation time of ions by secondary mass spectrometry was 50 ms. The impact chamber energy (high energy collision induced dissociation, HCD) was set at 28 eV for all precursor ions, and the dynamic exclusion was set at 25 s.

### Expression, purification, and functional assay of the oxidoreductase Oxr-1 of strain GUIA.

Two primers (Table S2) were used for amplification of the encoding gene of Oxr-1 (accession no. CP094930) from strain GUIA. The PCR products were cut with corresponding enzymes and ligated to pGEX-4T-1 cut by same above enzymes, resulting in the expression vector of *oxr-1*. The vector was then transformed into E. coli BL21(DE3) strain for overexpression. E. coli BL21(DE3) cells were incubated at 37°C for 2 to 3 h until the OD_600_ reached about 0.6 to 0.8. Then, Isopropyl β-D-1-thiogalactoside (IPTG) with a final concentration of 0.2 mM was added into the culture. The culture was then transferred to 16°C and incubated for another 12 h. Cells were harvested by centrifugation at 8,000 *g* for 20 min and resuspended in an appropriate lysis buffer A (20 mM HEPES, 300 mM KCl, 1 mM DTT, 10% glycerin, pH = 7.5). The lysates were treated with ultrasound on ice and the lysates were clarified by centrifugation on a Baker Ti-45 rotor at 12,000 *g* for 60 min. The supernatants were filtered by polyethersulfone membranes with a pore size of 0.22 μm (Merck Millpore, USA). The clarified lysate was loaded on a GSTrap HP column (GE Healthcare, USA) and the GST-tagged proteins (or GST alone) were eluted on an AKTA purifier system (Agilent, USA) by using a linear gradient with buffer B (20 mM HEPES, 300 mM KCl, 10 mM reduced glutathione, 1 mM DTT, 10% glycerol, pH = 7.5). Afterwards, we checked the purity of proteins by using SDS-PAGE assay. To check the degradation capability of GST or GST-Oxr-1, 600 μL of waterborne polyurethane was added per 100 mL of LB broth, and 900 μL of the final solution was dispensed into a sterile 1.5 mL centrifuge tube. Afterwards, GST or GST-Oxr-1 at the final concentration of 1.25 mg/mL was added to the reaction system and incubated for 24 h at 37°C. The change in OD_400_ was tested before and after the reaction (24). LB broth was used as the negative control. Three replicates for each treatment were performed.

### Fourier transform infrared analysis.

The waterborne polyurethane was treated by GST or GST-Oxr-1 as described above. Then the reaction products were precipitated by centrifugation at 3,000 *g*. The precipitation was collected, dried at room temperature, and measured by FTIR. On the other hand, the sterile PBAT film (type GB/T 38082-2019, 0.035 mm in thickness) was treated by GST or GST-Oxr-1 as described above for waterborne polyurethane at 37°C for 24 h. The film was then sonicated, washed, dried, and subjected to FTIR analysis (58). After air drying, GST or GST-Oxr-1 treated plastic films were recorded over the wavelength range of 450 to 4,000 cm^−1^ at a resolution of 1 cm^−1^ using a Nicolet-360 FTIR (Waltham, USA) spectrometer operating in ATR mode (59). Thirty-two scans were taken for each spectrum.

### Scanning electron microscopy observation.

To evaluate the degradation effect of PBAT film (type GB/T 38082-2019, 0.035 mm in thickness) by GST or GST-Oxr-1 at a final concentration of 3.5 mg/mL, the films were treated at 37°C for 24 h and observed by SEM (Hitachi S-3400 N, Japan). After treatment, the PBAT films were washed in ultrasonic cleaner with 1% SDS, distilled water, and then 75% ethanol (60), and then were air-dried. Dried specimens were sputter coated for 5 min with gold and platinum (10 nm) using a Hitachi MC1000 Ion Sputter (Japan). Observation was done using a field emission SEM operating at an accelerating voltage of 5 kV.

### Data availability.

Complete genome sequences of B. velezensis GUIA have been deposited at GenBank under the accession number CP094930. Raw sequencing reads for transcriptomic analysis have been deposited at NCBI (accession number: PRJNA901673).
