# Supplementary material for: A Deep-Sea Bacterium Is Capable of Degrading Polyurethane
Source: Microbiol Spectr. 2023 Mar 30;11(3):e00073-23. doi: 10.1128/spectrum.00073-23 (PMC10269918; doi:10.1128/spectrum.00073-23)
Supplement: Supplemental file 1 — Supplemental material. Download spectrum.00073-23-s0001.pdf, PDF file, 0.8 MB [file spectrum.00073-23-s0001.pdf]

SUPPLEMENTAL INFORMATION

A deep-sea bacterium is capable of degrading polyurethane

Zhi Gui[1], Guangchao Liu[1], Xin Liu[1], Ruining Cai[2,3,4], Rui Liu[2,3,4], Chaomin Sun[2,3,4*]

[1]Key Lab of Plant Biotechnology in Universities of Shandong Province, College of Life Science, Qingdao Agricultural University, Qingdao 266109, China.

[2]CAS and Shandong Province Key Laboratory of Experimental Marine Biology & Center of Deep Sea Research, Institute of Oceanology, Chinese Academy of Sciences, Qingdao, China.

[3]Laoshan Laboratory, Qingdao, China.

[4]Center of Ocean Mega-Science, Chinese Academy of Sciences, Qingdao, China.

[*] Corresponding author

Chaomin Sun Tel.: +86 532 82898857; fax: +86 532 82898857.

E-mail address: sunchaomin@qdio.ac.cn

Table S1 Genomic information of *B. velezensis* GUIA

Genomic information	Sequencing analysis
**NCBI accession number**	CP094930
**Total genome sequence length（bp）**	3929584
**Total number of genes annotated**	4,024
**The total length of the annotated gene（bp)**	3,526,305
**G+C content（%）**	46.5
**Number of chromosomes**	1

Table S2 Primers used for vector construction

Primer name	Primer sequence
**501 F**	5’-ATGGGATCAGCACAGTTAAC-3’
**501 R**	5’-TTTATACGATGGCGGGATGC-3’
**501 4T-1 F**	5’-GCATCCCGCCATCGTATAAACTCGAGCGGCCGCATCGTGA-3’
**501 4T-1 R**	5’-GTTAACTGTGCTGATCCCATGGATCCACGCGGAACCAGAT-3’

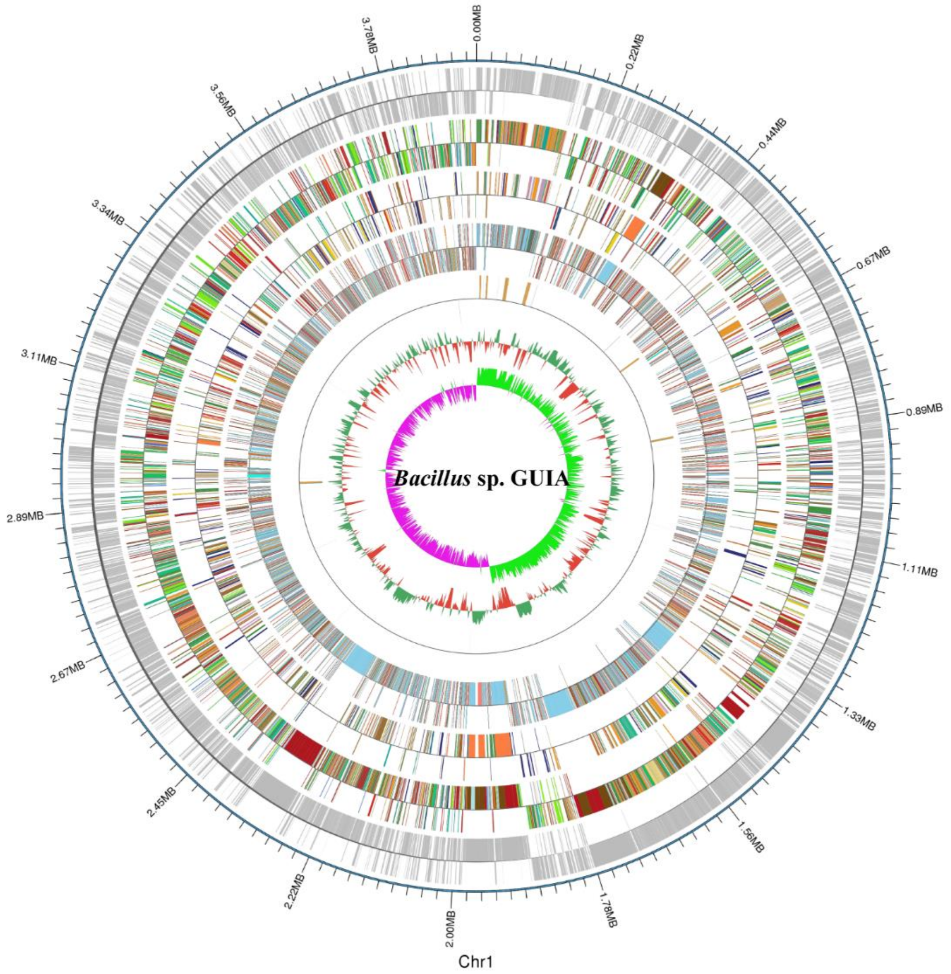

Fig. S1 Circular diagram of the genome of *B. velezensis* GUIA. The outermost circle is the position coordinates of the genome sequence. From the outside to the inside, it is respectively the coding gene, the result of gene functional annotation, ncRNA and the GC content of the genome. The inward red part indicates that the GC content in this region is lower than the average GC content in the whole genome, while the outward green part is the opposite.

A

Protein (weight per vial)	Molecular weight (M_r)	Source
Ovalbumin (50 mg)	43 000	Hen egg
Conalbumin (50 mg)	75 000	Chicken egg white
Aldolase* (50 mg)	158 000	Rabbit muscle
Ferritin* (15 mg)	440 000	Horse spleen
Thyroglobulin (50 mg)	669 000	Bovine thyroid
Blue dextran 2000 (50 mg)	2 000 000	

* These proteins are supplied mixed with sucrose or mannitol to maintain stability and aid their solubility.

B

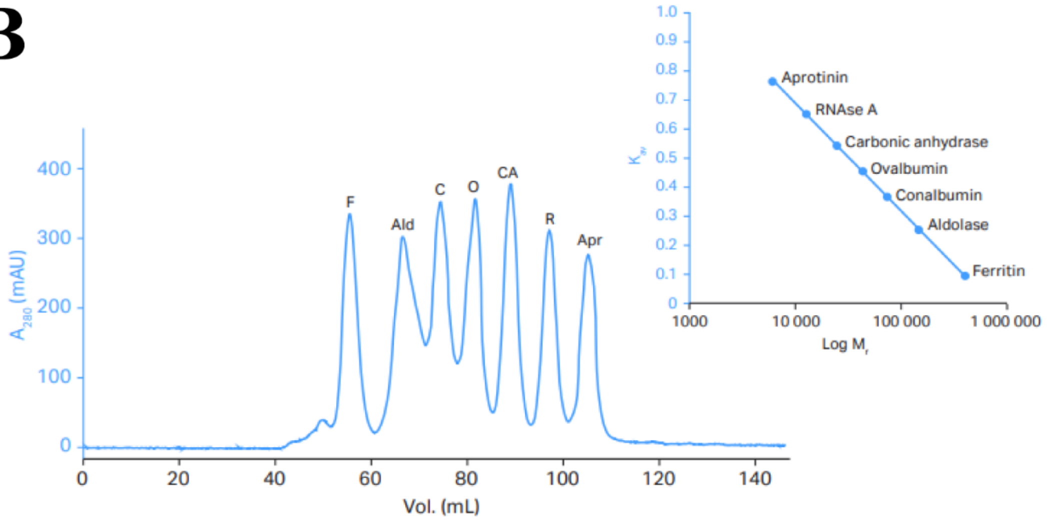

Fig. S2 Gel filtration calibration for HiLoad 16/600 Superdex 200 pg column. (A) Standard proteins and molecular weight used for the calibration of HiLoad 16/600 Superdex 200 pg column. (B) Chromatographic separation and calibration curve for the standard proteins of HiLoad 16/600 Superdex 200 pg column.
